# Protein oxidation in the intermembrane space of mitochondria is substrate-specific rather than general

**DOI:** 10.15698/mic2014.01.130

**Published:** 2014-03-03

**Authors:** Valentina Peleh, Jan Riemer, Andrew Dancis, Johannes M. Herrmann

**Affiliations:** 1 Cell Biology, University of Kaiserslautern, Erwin-Schrödinger-Strasse 13, 67663 Kaiserslautern, Germany.; 2 Cellular Biochemistry, University of Kaiserslautern, Erwin-Schrödinger-Strasse 13, 67663 Kaiserslautern, Germany.; 3 Division of Hematology-Oncology, Department of Medicine, University of Pennsylvania, 421 Curie Blvd., Philadelphia PA 19104, USA.

**Keywords:** cysteine oxidation, disulfide bonds, Dre2, Fe-S clusters, Mia40, mitochondria, oxidative protein folding

## Abstract

In most cellular compartments cysteine residues are predominantly reduced. However, in the bacterial periplasm, the ER and the mitochondrial intermembrane space (IMS), sulfhydryl oxidases catalyze the formation of disulfide bonds. Nevertheless, many IMS proteins contain reduced cysteines that participate in binding metal- or heme-cofactors. In this study, we addressed the substrate specificity of the mitochondrial protein oxidation machinery. Dre2 is a cysteine-rich protein that is located in the cytosol. A large fraction of Dre2 bound to the cytosolic side of the outer membrane of mitochondria. Even when Dre2 is artificially targeted to the IMS, its cysteine residues remain in the reduced state. This indicates that protein oxidation in the IMS of mitochondria is not a consequence of the apparent oxidizing environment in this compartment but rather is substrate-specific and determined by the presence of Mia40-binding sites.

## INTRODUCTION

Mitochondria contain two aqueous subcompartments, the matrix and the intermembrane space (IMS). The redox properties of these compartments are presumably very different: the matrix, like the cytosol of bacteria or eukaryotes, contains thioredoxin and glutaredoxin systems which keep cysteine residues reduced at steady state levels 1,2,3. In contrast, the IMS contains a dedicated machinery for the introduction of disulfide bonds into proteins 4,5,6,7. Thus far, more than 20 IMS proteins have been identified that contain structural disulfide bonds, and the list is steadily growing 8,9,10,11. Nevertheless, many proteins and protein domains of the IMS contain reduced cysteine residues, and these serve as coordination sites for metal ions (e.g. Cox2, Cox11, Cox17, Sco1, Sco2), iron-sulfur clusters (e.g. the Rieske iron sulfur protein Rip1) or heme cofactors (e.g. cytochrome *c*, cytochrome *c_1_*) 4. Thus, it is possible that disulfide bonds are formed in some IMS proteins and not in others. Alternatively disulfide bonds may be formed in all IMS proteins and then reduced by special reductases so that cofactors may be inserted. For example, in the bacterial periplasm, the reducing enzymes CcmH and CcmG open disulfide bonds in apocytochrome *c* to allow its binding to heme 12.

The mitochondrial disulfide relay consists of the oxidoreductase Mia40 and the sulfhydryl oxidase Erv1. Mia40 directly binds to newly synthesized IMS proteins and promotes their translocation from the cytosol into the IMS 5,13,14,15,16. Mia40 forms a hydrophobic binding cleft that interacts with short stretches of the imported substrates which have been referred to as MISS (mitochondrial IMS-sorting signal) or ITS (IMS targeting signal) sequences 17,18,19. These sequences do not follow a strict consensus and are characterized by two or three hydrophobic residues in proximity to a cysteine residue.

Most substrates of Mia40 that were identified so far are of simple structure and contain four cysteine residues that are present in twin Cx_3_C or twin Cx_9_C arrangements. Recently, a few more complex substrates were identified which contain nine (yeast Dre2) or ten (anamorsin, the human homologue of the Dre2 protein, and yeast Atp23) cysteine residues 20,21. Atp23 is a protease of the IMS that plays a role in the biogenesis of the F_o_F_1_-ATPase complex of the inner membrane 22,23. Anamorsin/Dre2, on the other hand, is a component of the cytosolic iron sulfur (Fe-S) assembly machinery 24, and its interaction with Mia40 in the IMS was therefore unexpected. However, studies in yeast cells reported that a fraction of Dre2 was associated with mitochondria which was particularly pronounced when Dre2 was overexpressed 25. NMR studies showed that purified Mia40 was able to oxidize anamorsin *in vitro* 21, thereby introducing two disulfide bonds into a domain which was previously suggested to serve as a Fe-S-binding region 25,26. From this it was concluded that anamorsin/Dre2 was the first identified Fe-S protein imported into the IMS, giving rise to speculation that it plays a role in cytosolic Fe-S biogenesis even when it is trapped in mitochondria.

In this study, we carefully analyzed the localization of Dre2 in yeast cells. We could confirm the association of Dre2 with mitochondria. However, our results indicate that Dre2 is tightly associated with the cytosol-exposed surface of the outer membrane of mitochondria but not present in the IMS. In its outer membrane-bound form it is partially resistant against protease. Furthermore, Dre2 does not contain disulfide bonds nor is its mitochondrial association influenced by the presence or absence of Mia40. Interestingly, even if Dre2 is artificially targeted to the IMS by fusion with an IMS-targeting signal, the nine cysteine residues in Dre2 remain reduced. However, upon addition of chemical oxidants such as diamide the *ims*Dre2 variant was found to be oxidized. Thus, the Mia40-dependent oxidation of proteins in the IMS only takes place in specific proteins and presumably relies on the presence of Mia40-binding sites.

## RESULTS

### Dre2 is a cytosolic protein associated with mitochondria

**Figure 1:**
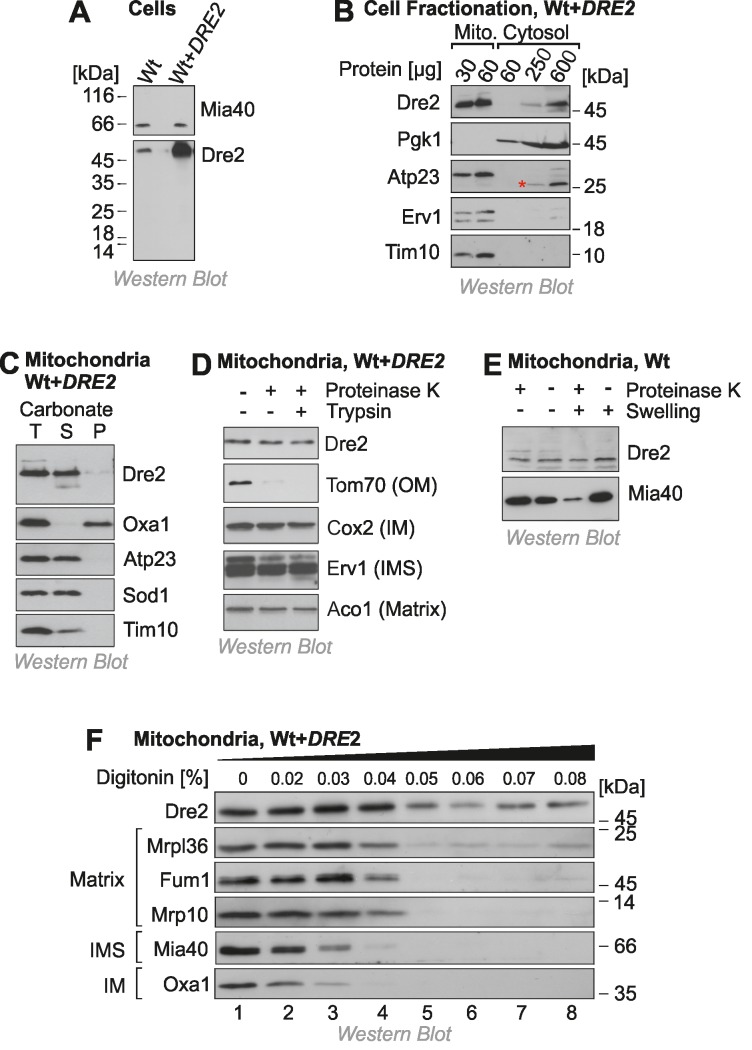
FIGURE 1: A fraction of Dre2 is associated with mitochondria. **(A)** Wild type (Wt) and Dre2-overexpressing cells (0.5 ODs) were lysed and the levels of Dre2, and of Mia40 for control, were detected by Western blotting. **(B)** Mitochondrial and cytosolic fractions were isolated from Dre2-overexpressing cells and analyzed by Western blotting. The asterisk indicates a crossreaction of the Atp23 antibody. **(C)** Mitochondria (50 μg) isolated from Dre2-overexpressing cells were either directly loaded to the gel (T, total) or incubated with 0.1 M Na_2_CO_3 _for 30 min on ice before separation of soluble (S) and membrane (M) fractions by centrifugation for 30 min at 100,000 x g. The integral membrane protein Oxa1 and the soluble or membrane-associated proteins Atp23, Sod1, Tim10 were used as controls. **(D)** Mitochondria were isolated from Dre2-overexpressing cells and incubated for 20 min with or without proteinase K or trypsin (100 μg/ml). The levels of Dre2 and control proteins were assessed by Western blotting. Only the outer membrane (OM) protein Tom70 was sensitive to the protease treatment. IM, inner membrane. **(E)** Wild type mitochondria were incubated for 30 min with protease at isoosmotic or hypoosmotic (swelling) conditions. Whereas the IMS protein Mia40 was protease-accessible upon hypotonic rupturing of the outer membrane, Dre2 remained inaccessible. **(F)** Mitochondria of Dre2-overexpressing cells were incubated with increasing concentrations of the detergent digitonin and exposed to proteinase K. Mitochondria were reisolated and the levels of the indicated proteins were detected by Western blotting.

To identify the intracellular distribution of Dre2 we made use of an antibody which specifically reacts with Dre2 and which recognizes the protein both in wild type and in Dre2-overexpressing cells (Fig. 1A). It was reported before that a considerable fraction of Dre2 is located on or in mitochondria, particularly when Dre2 is overexpressed 25. We therefore fractionated yeast cells into a mitochondrial and a cytosolic fraction and analyzed these fractions by Western blotting (Fig. 1B). The cytosolic protein Pgk1 was exclusively found in the cytosolic fraction and the mitochondrial proteins Atp23, Erv1 and Tim10 in the mitochondrial fractions. In contrast, Dre2 was found both in the cytosolic and the mitochondrial fraction. Actually, 30 μg of mitochondrial protein showed a much more intensive Western blot signal for Dre2 than 600 μg of cytosolic protein. If one considers the much larger volume of the cytosolic fraction obtained in the experiment, we estimate that 40-50% of the total Dre2 protein in the overexpression strain coisolated with purified mitochondria.

Next, we analyzed the submitochondrial location of Dre2. First we tested the fractionation of Dre2 by alkaline extraction. In contrast to the integral membrane protein Oxa1, mitochondrial Dre2 was released from membranes when mitochondria were treated with carbonate (Fig. 1C). This indicates that Dre2 is either associated with membrane surfaces or resides as soluble protein within mitochondria. To assess the localization of Dre2 further, we incubated isolated mitochondria with proteases like proteinase K or trypsin (Fig. 1D). Protease treatment did not reduce the amounts of Dre2, indicating that the protein either resides inside the mitochondria or has a protease-resistant nature when associated with mitochondria.

To further analyze the association of Dre2 with mitochondria, we subfractionated mitochondria by hypotonic swelling which specifically opens the outer membrane. However, swelling did not increase the protease-sensitivity of Dre2 as it did for the IMS protein Mia40 (Fig. 1E). Thus, mitochondrial Dre2 is either in the matrix or it exhibits protease-resistance outside the matrix. To distinguish between these possibilities, we incubated mitochondria with increasing amounts of digitonin in the presence of proteinase K. Whereas low concentrations of digitonin open the outer membrane, the inner membrane is only lysed upon incubation with concentrations larger than 0.04% (Fig. 1F). In this assay Dre2 behaved differently from other mitochondrial proteins, and, even upon complete lysis of mitochondrial membranes, a significant fraction of Dre2 remained protease-resistant. This suggests that Dre2 in mitochondria is rather resistant to protease, particularly if membranes are not lysed with detergent.

### Dre2 is bound to the surface of mitochondria

**Figure 2:**
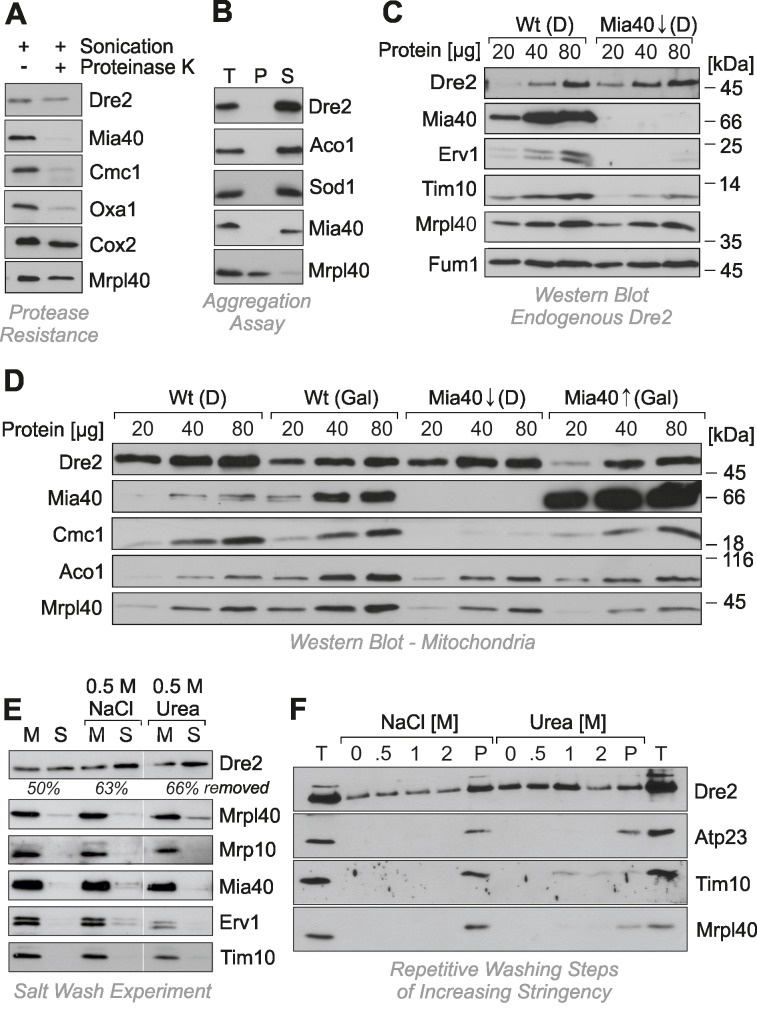
FIGURE 2: Dre2 is not in but on mitochondria. **(A)** Mitochondria of Dre2-overexpressing cells were sonicated in the presence of 50 μg/ml proteinase K so that only intrinsically protease-stable proteins remain undigested. Proteins were collected by TCA-precipitation and visualized by Western blotting. Cox2 and Mrpl40 are protease-resistant due to their integration into large protein complexes. **(B)** Mitochondria of Dre2-overexpressing cells were lysed with 1% Triton X-100. The extract was either directly loaded to the gel (T, total) or after separation of supernatant (S) and pellet (P) fractions by centrifugation for 30 min at 140,000 x g. **(C)** Mitochondria were isolated from wild type or *GAL-MIA40* cells. The levels of endogenous Dre2 and control proteins were analyzed by Western blotting. **(D)** Wild type or *GAL-MIA40* cells were transformed with a Dre2-overexpression plasmid and grown in glucose (D) or galactose (Gal) containing medium. Mitochondria were isolated and subjected to Western blotting with antibodies against the depicted proteins. **(E)** Mitochondria were isolated from a Dre2-overexpressing strain and incubated with SEH buffer (0.6 M sorbitol, 5 mM EDTA, 20 mM Hepes pH 7.4) in the absence or presence of 0.5 M sodium chloride or urea for 10 min at 30°C. Mitochondria were reisolated by centrifugation. Proteins from the mitochondrial (M) and supernatant (S) fraction were collected by TCA precipitation. The Dre2 signals were quantified and the proportion of Dre2 is indicated that was removed from the mitochondria in the washing step. **(F)** Mitochondria of Dre2-overexpressing cells were incubated in SEH buffer for 10 min at 30°C. Mitochondria were reisolated by centrifugation for 7 min at 16,000 x g and dissolved in SEH buffer containing 0.5 M NaCl or urea, respectively. Incubation and centrifugation steps were repeated with increasing concentrations of salt or urea as indicated. Proteins from the supernatant was precipitated by TCA. P, mitochondrial pellet after all washing steps. T, total representing 50 μg mitochondria that were directly loaded.

To confirm the protease resistance of Dre2, mitochondria were sonicated in the presence of proteinase K so that the protease had access to all mitochondrial compartments (Fig. 2A). Even under these harsh conditions, Dre2 remained stable and was not degraded. Hence mitochondrial Dre2 exhibited protease-resistance, making protease protection assays problematic for assessing the location of Dre2. Since the association with mitochondria was strongly increased upon overexpression of Dre2 25, we tested whether the overexpressed proteins forms aggregates. To this end, we lysed isolated mitochondria from Dre2-overexpressing cells with Triton X-100 and isolated large protein particles by centrifugation for 30 min at 140,000 x g (Fig. 2B). Thereby, Dre2 completely remained in the supernatant suggesting that the protein does not form aggregates.

It was concluded from *in vitro* experiments that the human Dre2 homolog anamorsin is a substrate of Mia40. We therefore tested whether the association of Dre2 with mitochondria was influenced by Mia40 levels (Fig. 2C). Mia40 is an essential protein so that Mia40 deletion mutants are not viable. Therefore we made use of a yeast strain in which the *MIA40* gene was under control of a regulatable *GAL10* promoter 27. Depletion of Mia40 from mitochondria resulted in the absence (Erv1) or strong reduction (Tim10) of its substrates. However, we did not observe considerably altered amounts of endogenous Dre2 in Mia40-depleted mitochondria (Fig. 2C).

To exclude that the residual traces of Mia40 in the Mia40-depletion strain are still sufficient for the recruitment of the relatively small amounts of endogenous Dre2 to mitochondria, we tested the relevance of Mia40 for mitochondrial association of overexpressed Dre2 (Fig. 2D). In wild type and *GAL*-*MIA40* cells the presence of glucose increased the levels of Dre2 in the mitochondrial fractions by roughly twofold. However, there was no striking difference between wild type and *GAL*-*MIA40* samples. Hence, the observed alterations are due to the change in the carbon source but are not considerably affected by the presence or absence of Mia40. This shows that Mia40 is not required for the association of Dre2 with mitochondria.

The protease resistance of mitochondrial Dre2 prompted us to test whether Dre2 might actually be not in the mitochondria but rather associated with its surface. We therefore tested whether Dre2 could be washed away from mitochondria (Fig. 2E). Indeed when isolated mitochondria were washed with buffer, a considerable fraction of Dre2 was released from the mitochondria. This fraction increased when 0.5 M sodium chloride or urea was added to the buffer. Under the same conditions, proteins of the IMS and the matrix remained in the mitochondrial pellet, confirming that mitochondrial membranes were not ruptured. The surface-associated salt-sensitive nature of Dre2 was particularly obvious, when mitochondria were washed repetitively with buffer of increasing concentrations of sodium chloride or urea. As shown in Fig. 2F, more and more of the mitochondria-associated Dre2 was released into the supernatant, whereas proteins of the IMS (Atp23, Tim10) or the matrix (Mrpl40) remained in the mitochondrial pellet unless high urea concentrations opened the outer membrane.

In summary, we conclude that Dre2 is not localized in the IMS of mitochondria, although we cannot exclude that a minute amount of the protein is present within mitochondria. However, there exists a considerable fraction of Dre2 that is tightly associated with the cytosol-exposed surface of mitochondria. This fraction is largely protease-resistant but can be released by detergent or high salt.

### Dre2 does not contain disulfide bonds *in vivo*

**Figure 3:**
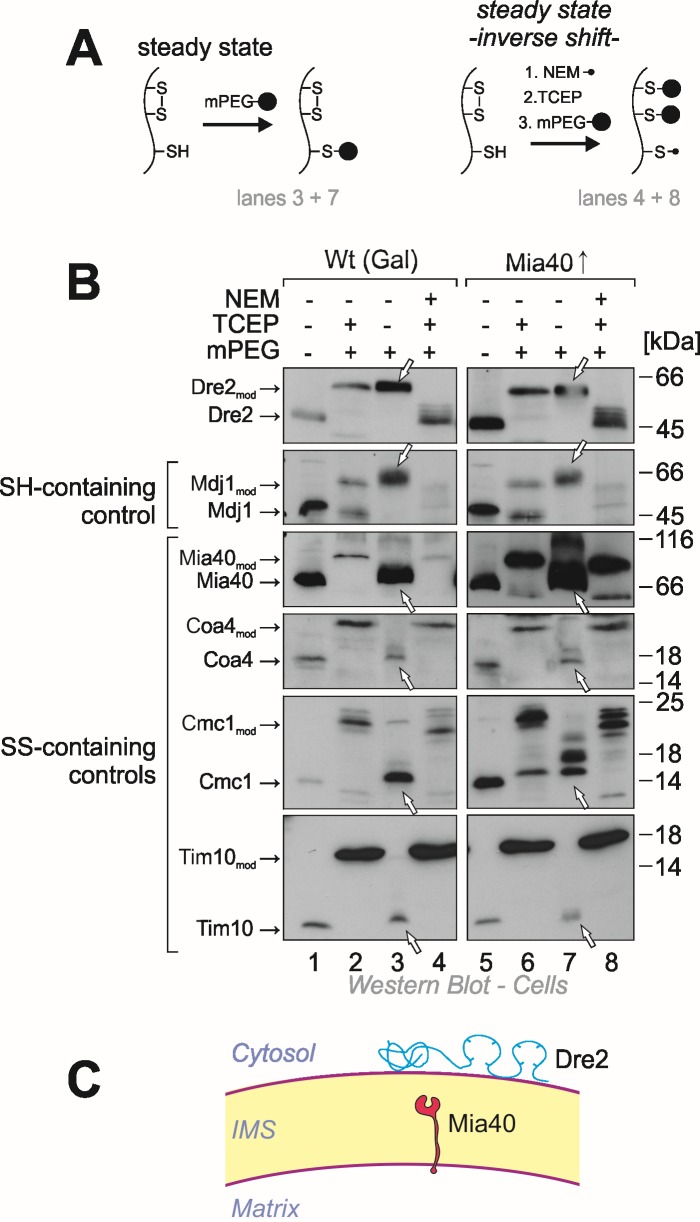
FIGURE 3: The cysteine residues in Dre2 are reduced at steady state conditions. **(A)**Scheme showing the modifications of reduced and oxidized protein thiols by treatment with the alkylating agents mPEG and NEM and the reductant TCEP. **(B)**Wild type or Mia40-upregulated cells in which Dre2 was overexpressed were harvested, acid treated with TCA to preserve the redox state of thiol groups and incubated with the indicated chemicals in subsequent reactions. Lanes 1 and 5 show non-modified proteins thereby serving as size standards for completely oxidized proteins. Lanes 2 and 6 show complete alkylation with mPEG-24 and hence the size expected for proteins in which all cysteines are reduced. Lanes 3 and 7 show the steady state of the proteins in the cell, lanes 4 and 8 the inverse shift after blocking thiols with NEM and opening of disulfides with TCEP. Arrowheads point at the signals of the steady state species which indicate that all thiols in Dre2 are present in the reduced form. **(C)** Schematic representation of Dre2 on the mitochondrial surface. Reduced cysteine residues are indicated. Our observations suggest that Dre2 does not contain structural disulfide bonds.

Yeast Dre2 and mammalian anamorsin share a conserved cysteine-rich domain which was proposed to bind an [4Fe-4S]-cluster 25,26 or, alternatively, to form structural disulfide bonds 21. The hypothesis that anamorsin contains disulfide bonds was purely based on *in vitro* experiments using purified protein. In order to test whether Dre2 contains disulfide bonds *in vivo*, we employed an alkylation assay that is well established in the field (Fig. 3A, left panel). For this assay, whole cells were treated with trichloroacetic acid (TCA), since protonation of the thiolate group at low pH prevents post-lysis protein oxidation. The extract was then directly treated with methyl-PEG-24-maleimide (mPEG-24), alkylating reduced thiols and thereby adding a mass of roughly 1.2 kDa per moiety. Hence only the size of proteins containing reduced thiols is shifted when mPEG-24-treated. Additionally, we carried out an "inverse shift" experiment in which reduced thiols were initially blocked by N-ethylmaleimide (NEM), and then disulfide bonds were opened with the thiol-free reductant tris(2-carboxyethyl)phosphine (TCEP) before mPEG-24 was added (Fig. 3A, right panel).

As shown in Fig. 3B, all thiol groups in Dre2 were accessible to mPEG-24, and the protein was completely shifted (lane 3) like in the "maximum shift" control in which all potential disulfides were initially reduced with TCEP (lane 2). Consistently, Dre2 remained inaccessible to mPEG-24 in the inverse shift samples (lane 4, compare to lane 1). Dre2 also remained completely reduced in Mia40-upregulated mitochondria. In the band pattern, Dre2 resembled other proteins with reduced cysteines such as the matrix DnaJ-homolog Mdj1, a zinc finger protein. Dre2 was different from proteins containing structural disulfide bonds such as Mia40, Coa4, Cmc1 or Tim10 (Fig. 3B, lower panels). From this we conclude that mitochondria-associated Dre2 does not contain structural disulfides, consistent with a role of the cysteine residues in Fe-S cluster binding (Fig. 3C).

### Dre2 can be artificially targeted to the IMS by fusion to a sorting sequence

**Figure 4:**
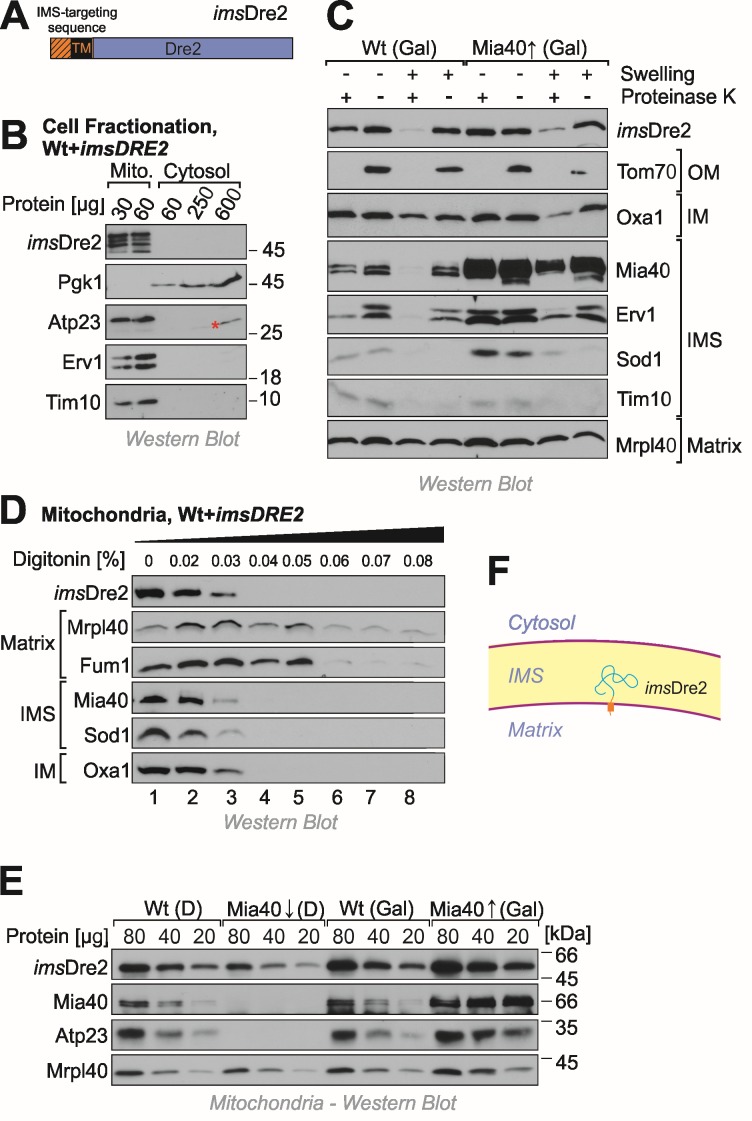
FIGURE 4: Dre2 can be directed into the IMS when fused to an IMS-targeting presequence. **(A)**Schematic structure of the *ims*Dre2 fusion protein. TM, transmembrane domain. **(B)**Cellular fractionation of *ims*Dre2-overexpressing wild type cells. Some degradation of *ims*Dre2 is visible due to the long exposure time used for this Western blot experiment. The asterisk indicates a crossreaction of the Atp23 antibody. **(C)***ims*Dre2 was expressed in wild type or Mia40-upregulated cells. Mitochondria were isolated and fractionated by hypotonic swelling as described in Fig. 1E. **(D)** Digitonin treatment of isolated mitochondria as described in Fig. 1F showing that *ims*Dre2 behaves like an IMS protein. **(E)** Steady state levels of *ims*Dre2 and control proteins were analyzed by Western blotting of mitochondria isolated from the indicated strains. **(F)** Scheme of the topology of the *ims*Dre2 fusion protein in mitochondria.

Next we decided to target Dre2 to the mitochondrial IMS by use of an N-terminal targeting sequence. To this end, we fused the entire Dre2 sequence to the IMS-targeting sequence of Mia40 (IMS-targeted Dre2, *ims*Dre2, Fig. 4A). Cellular fractionation experiments confirmed the targeting of the *ims*Dre2 protein to mitochondria and not even traces of this protein were detectable in the cytosol (Fig. 4B).

Next we overexpressed *ims*Dre2 in wild-type and *GAL-MIA40 *cells. Mitochondria were isolated and the location of *ims*Dre2 was determined by subfractionation of the mitochondria (Fig. 4C). Since the levels of *ims*Dre2 were much higher than those of the endogenous Dre2, which was still present in these cells, we could specifically detect the *ims*Dre2 protein by Western blotting under the conditions used in these experiments. *ims*Dre2 was inaccessible to protease in mitochondria but became accessible when the outer membrane was ruptured by hypotonic swelling. Due to the presence of a transmembrane domain in the Mia40 presequence, the *ims*Dre2 protein was not released from mitoplasts, whereas soluble IMS proteins like Sod1 or Tim10 were not found in mitoplasts even when no protease was present.

Proper localization of *ims*Dre2 in the IMS was also confirmed by digitonin fractionation where the fusion protein became protease-accessible at similar digitonin concentrations as other IMS proteins such as Mia40 or Sod1 (Fig. 4D).

The Mia40 presequence used for the construction of the *ims*Dre2 protein mediates protein import via the presequence pathway which does not depend on Mia40 activity. Hence, Mia40 was dispensable for the import of *ims*Dre2 and *ims*Dre2 was still found in mitochondria isolated from Mia40-depelted cells whereas Mia40 substrates (Atp23) were not detectable (Fig. 4E). From this we conclude that *ims*Dre2 is targeted into the IMS of mitochondria where it is tethered to the inner membrane by its N-terminal membrane anchor (Fig. 4F).

### *ims*Dre2 remains reduced *in vivo*

**Figure 5:**
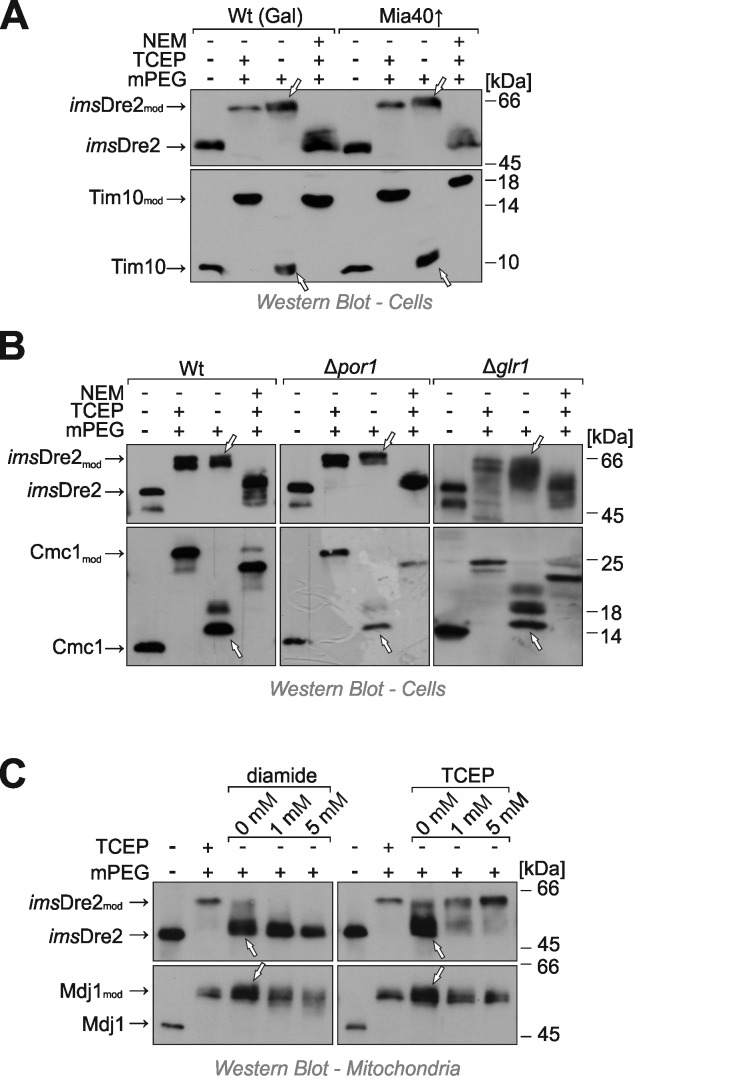
FIGURE 5: Despite its location in the IMS, *ims*Dre2 does not contain disulfides. **(A, B)** The redox state of disulfides in *ims*Dre2 and control proteins was analyzed in wild type, Δ*por1* and Δ*glr1* cells as described in Fig. 3. **(C)** Mitochondria were isolated from *ims*Dre2-expressing cells, incubated in the presence or absence of diamide or TCEP. Proteins were TCA-precipitated before their redox state was analyzed as described before.

Next we tested the redox state of the *ims*Dre2 protein by use of the mPEG shift assay explained above. Yeast cells expressing the fusion protein were grown to log phase, harvested and treated with TCA to preserve the redox state of protein thiols. Extracts were prepared and treated with NEM, TCEP or/and mPEG-24. Interestingly, when *ims*Dre2 was incubated with mPEG-24 (Fig. 5A, lane 3), the protein was shifted to the same size observed with the fully alkylated "maximum shift" control (Fig. 5A, lane 2). Consistently, when the samples were first treated with NEM, then reduced by TCEP and subsequently exposed to mPEG-24, *ims*Dre2 was not shifted. From this we conclude that the thiol groups in the *ims*Dre2 are all accessible and hence reduced. The same was seen in cells in which Mia40 was overexpressed, indicating that *ims*Dre2 was not oxidized by Mia40 or, alternatively, was rapidly reduced after initial oxidation.

Reduced glutathione plays a crucial role in counteracting protein oxidation in the IMS 2,3,28. The reducing conditions in the IMS depend on the porin (Por1) channels in the outer membrane which allow the equilibration of the glutathione pools of the cytosol and the IMS, and on the cytosolic glutathione reductase Glr1, which reduces oxidized glutathione by consumption of NADPH 1,3. In the Δ *por1* mutant as well as in Δ *glr1 *cells, the *ims*Dre2 was still found to be reduced at steady state levels (Fig. 5B), suggesting that *ims*Dre2 is not oxidized rather than being rapidly reduced by glutathione after its initial oxidation.

The observation that *ims*Dre2 remained reduced despite its location in the IMS might simply reflect the inability of its cysteine residues to form disulfide bonds. Therefore we tested whether the cysteine residues in *ims*Dre2 can be oxidized *in vitro*. We isolated mitochondria from *ims*Dre2-expressing cells and incubated them for 15 min with the chemical oxidant diamide. As shown in Fig. 5C, upon exposure to these conditions, most cysteine residues in *ims*Dre2 became inaccessible to mPEG-24, unless they were first reduced by TCEP. Moreover, we observed that *ims*Dre2 was oxidized in isolated mitochondria even without diamide treatment - unlike in TCA precipitates of whole cells - indicating that disulfides are formed in *ims*Dre2 already during mitochondrial preparation (Fig. 5C). This is consistent with previous observations by us that the isolation of mitochondria from yeast cells can lead to the oxidation of thiol groups in mitochondrial proteins (not shown). Thus, we conclude that in isolated mitochondria *ims*Dre2 became oxidized, forming several intramolecular disulfides. However, as long as cells were intact, no oxidation of *ims*Dre2 in the IMS was observed.

### *ims*Dre2 does not interact with Mia40 after its import into the IMS

**Figure 6:**
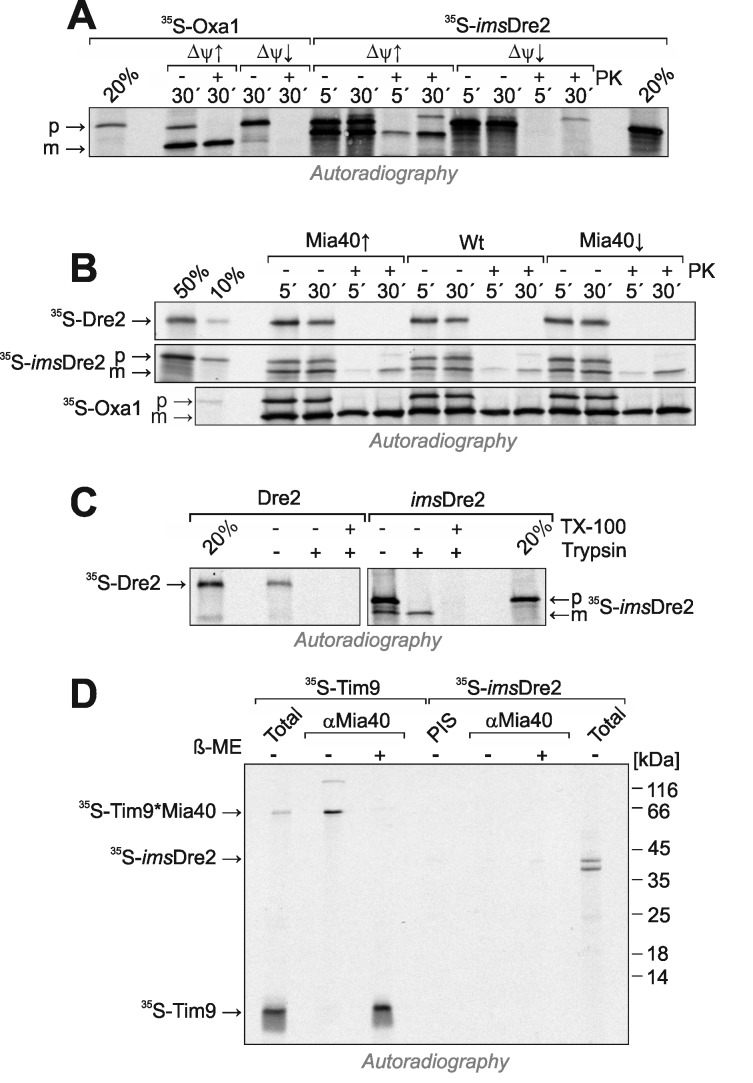
FIGURE 6: *ims*Dre2, but not Dre2, can be imported into isolated mitochondria. **(A)**Oxa1 and *ims*Dre2 were synthesized in reticulocyte lysate in the presence of ^35^S-methionine. The proteins were either directly loaded onto the gel (20%) or incubated with isolated wild type mitochondria in the absence (Δ ψ↑) or presence (Δ ψ ↓) of valinomycin. Subsequently, mitochondria were incubated with or without proteinase K (PK) to remove non-imported material, reisolated and dissolved in Laemmli buffer. Precursor (p) and mature (m) forms of the proteins are indicated. **(B)**Import reactions were carried out with Dre2, *ims*Dre2 and Oxa1 using mitochondria of the indicated strains. **(C)** Import reactions with wild type mitochondria were carried out with urea-denatured radiolabeled Dre2 and *ims*Dre2 using the conditions described by the Banci and colleagues 21. **(D)**Radiolabeled Tim9 or *ims*Dre2 were imported into isolated mitochondria for 2 min. Mitochondria were treated with 150 mM NEM, lysed and either directly loaded on the gel (Total) or subjected to immunoprecipitation with Mia40-specific antibodies (αMia40) or preimmune serum (PIS). To preserve and dissociate disulfides, sample buffer without or with β-mercaptoethanol (β-ME) was used.

*In vitro* import experiments with radiolabeled preproteins have proved to be a powerful method to analyze the biogenesis of mitochondrial proteins. We used this approach to follow the import pathways of Dre2 and *ims*Dre2, and to compare their import to that of the well characterized inner membrane protein Oxa1. First, we synthesized *ims*Dre2 and Oxa1 in reticulocyte lysate in the presence of ^35^S-methionine and incubated these preproteins with wild-type mitochondria (Fig. 6A). Upon incubation of the Oxa1 precursor (p) with mitochondria, a faster migrating mature (m) form of Oxa1 was generated which was resistant to added protease. When the membrane potential was dissipated by addition of valinomycin (Δ ψ ↓), no processing of Oxa1 was observed and Oxa1 remained protease-accessible. Similarly, *ims*Dre2 was processed and imported into mitochondria in a membrane potential-dependent manner. Hence, mitochondrial import of the *ims*Dre2 fusion protein can be followed *in vitro*.

Next we also produced radiolabeled Dre2 and incubated it with mitochondria (Fig. 6B). In contrast to *ims*Dre2 and Oxa1, Dre2 was not imported into mitochondria and a protease-resistant species was not observed. Even when we used the same conditions described in Banci *et al.* 21, where the radiolabeled proteins were initially denatured with urea, we could not observe any protein import of Dre2 into mitochondria (Fig. 6C). When the levels of mitochondrial Mia40 were up- or down-regulated, the import efficiencies of Oxa1 or *ims*Dre2 were not influenced, confirming that Mia40 was not critical for their import (Fig. 6B).

The observation that Mia40 was not required for import of *ims*Dre2 does not necessarily exclude an interaction of both proteins. In order to directly test whether Mia40 forms mixed disulfides with imported *ims*Dre2, *ims*Dre2 and the Mia40 substrate Tim9 13,14 were imported into Mia40-upregulated mitochondria (Fig. 6D). Then mitochondria were lysed and Mia40 was isolated by immunoprecipitation. Subsequently, the samples were analyzed by SDS-PAGE under reducing or non-reducing conditions. Upon import of Tim9, a heterodimer of Tim9 and Mia40 was formed that could be isolated by Mia40 antibodies and dissolved by β-mercaptoethanol. In contrast, such a dimer was not formed with the imported *ims*Dre2, and no evidence indicated an interaction of Dre2 with Mia40. Hence, although *ims*Dre2 was imported into the IMS and contained nine cysteine residues, this protein did not interact with Mia40, pointing to a highly specific substrate profile of the mitochondrial disulfide relay.

### Recombinant Mia40 oxidizes Tim9 but not Dre2

**Figure 7:**
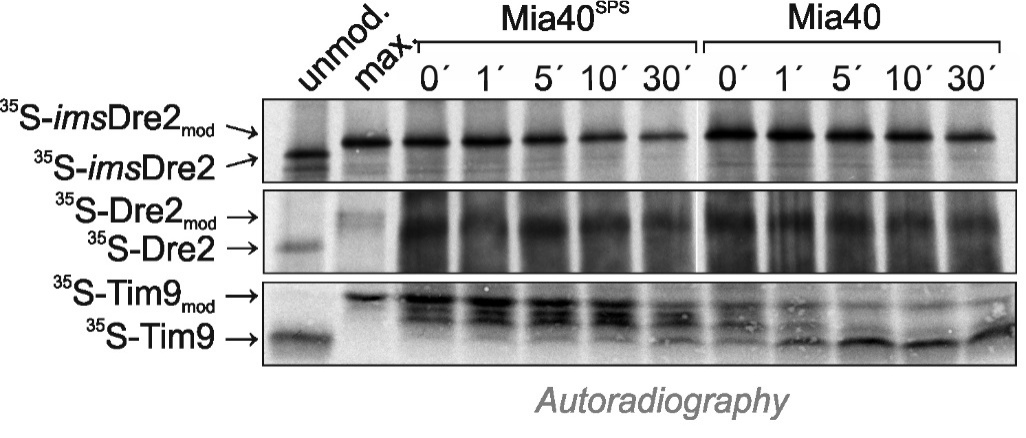
FIGURE 7: Tim9, but not Dre2, is oxidized by recombinant Mia40 *in vitro*. Radiolabeled *ims*Dre2, Dre2 and Tim9 were reduced and either directly loaded to the gel (unmodified, unmod.), or incubated with 35 μM Mia40^SPS^ or Mia40 for the times indicated, denatured with SDS and treated with 15 mM mPEG-12. For control, a sample of reduced protein was directly treated with mPEG-12 so that all thiols are alkylated (maximum shift, max.).

In the past, reconstituted assays with recombinant proteins were successfully used to follow the Mia40-mediated protein oxidation *in vitro* 20,28,29,30. We therefore purified recombinant Mia40 and a non-functional Mia40 variant that lacks the redox-active cysteine pair (Mia40^SPS^). These proteins were incubated with radiolabeled *ims*Dre2, Dre2 or Tim9 in which the cysteine residues had been reduced by TCEP treatment. After different time periods, the proteins were collected by TCA precipitation, and reduced thiols were modified with mPEG-12. As shown in Fig. 7, the migration behaviour of *ims*Dre2 and Dre2 in this assay did not change when incubated with Mia40 or Mia40^SPS^. In contrast, Tim9 was rapidly oxidized which resulted in a faster migrating species after the alkylation with mPEG-12. Again, this confirms our previous conclusion that Dre2 does not serve as substrate for Mia40, pointing to a considerable substrate specificity of the mitochondrial oxidoreductase Mia40.

## DISCUSSION

The human protein anamorsin and its yeast homolog Dre2 are essential components of the cytosolic Fe-S assembly machinery. It was proposed that a fraction of anamorsin/Dre2 resides in the IMS of mitochondria 25, where Mia40 would introduce two disulfide bonds into a C-terminal Cys-x_2-_Cys-x_7_-Cys-x_2_-Cys motif 21. Here we show that in baker’s yeast, Dre2 (i) is tightly associated with the cytosolic surface of mitochondria, (ii) contains only reduced thiol residues and no disulfide bonds, (iii) does not interact with Mia40 *in vivo* or *in vitro*, and (iv) is not oxidized when mixed with purified Mia40 *in vitro*. At this stage, we cannot formally exclude that there are differences between human anamorsin and yeast Dre2. However, the region around the four C-terminal cysteine residues that were proposed to be oxidized, is highly conserved and almost identical between animals and fungi (Fig. S1). Moreover, the function of Dre2 in the biogenesis of cytosolic Fe-S clusters 24,25 is not likely to be compatible with a localization in the IMS. Nevertheless, we could confirm a close association of Dre2 with mitochondria. Interestingly, the Dre2-interacting protein Tah18 was also reported to associate with mitochondria, at least upon stress conditions 31. It therefore appears conceivable that the mitochondrial association of the Dre2-Tah18 complex is of physiological relevance in its role of promoting the biogenesis of cytosolic Fe-S clusters.

We observed that the mitochondrion-associated Dre2 is rather resistant to proteases such as trypsin and proteinase K, unless the membranes of the organelle are lysed by detergents. The protein *per se* is not resistant to protease, and the protein produced in reticulocyte lysate is easily digested as is the *ims*Dre2. The explanation for the protease resistance of mitochondrial Dre2 can be that the properties of the protein are altered by its association with the mitochondrial outer membrane. It was shown before for other proteins such as the superoxide dismutase Sod1 that the binding to the outer membrane can render a protein inaccessible to proteases 32,33. This protease-stability of mitochondrial Dre2 poses a problem for interpretation of classical protease accessibility assays and presumably is the reason why initially it was suggested that a fraction of Dre2 is located within mitochondria 21,25. When *in vitro* synthesized Dre2 was incubated with mitochondria, a fraction of it bound to mitochondria (Fig. 6B, C), however, it remained protease-sensitive. Presumably this is due to the fact that the coordination of Fe-S clusters is a prerequisite for the proteolytic stability of Dre2.

Even when artificially directed into the IMS of mitochondria by use of an N-terminal targeting sequence, Dre2 remained reduced and did not interact with Mia40. This was unexpected because Dre2 contains nine cysteine residues, several of which are flanked by hydrophobic residues. The MISS/ITS signals that were shown to be both necessary and sufficient for the binding of IMS proteins to Mia40 do not match a strict consensus and it is still rather unclear how Mia40 recognizes its substrate proteins 16,17,18,19,34. The data presented in this study demonstrate that the formation of disulfide bonds in IMS proteins occurs with stringent substrate specificity. Although *ims*Dre2 was oxidized by diamide or by molecular oxygen during mitochondrial preparation, the protein remained reduced *in vivo*, even in the setting of Mia40 overexpression.

From early studies on the protein oxidation process in the ER it was proposed that disulfide formation is promoted by a chemical equilibrium with an "oxidizing milieu" established by a high ratio of glutathione-disulfide to reduced glutathione 35,36. Only with the identification of the sulfhydryl oxidase Ero1 37,38 did it became evident that protein oxidation is driven by members of the PDI family that are maintained in an oxidized state by Ero1 39,40 and other ER proteins 41,42,43. PDIs are efficient oxidoreductases that presumably oxidize most secretory proteins during or directly after their translocation into the ER. Similarly, in the bacterial periplasm, cysteine residues are rapidly oxidized by DsbA so that disulfide bonds are initially formed between consecutive cysteine pairs during protein translocation across the inner membrane; hence proteins that require reduced cysteine residues, e.g. for heme binding, need to be reduced again in a subsequent reductase-mediated reaction 44,45. The situation in the IMS might differ generally from the ER or the periplasm in that many proteins or protein domains in the IMS contain cysteine residues for the coordination of cofactors such as heme or copper 4. For example, the most abundant IMS protein, cytochrome *c*, contains two cysteine residues that bind the heme cofactor of the protein. A highly substrate-specific oxidation of cysteine residues by Mia40 might prevent non-specific oxidation of these thiols and an unwanted interference with the heme insertion process. It might be speculated that the DsbA system in the periplasm of bacteria was replaced by the Mia40-Erv1 system in the IMS of mitochondria due to its higher substrate specificity. It will be very interesting in the future to further explore how the different cysteine oxidation machineries of bacteria and eukaryotes specifically introduce disulfide bonds into their substrates.

## MATERIALS AND METHODS

### Yeast strains and media

Yeast strains were derived from the wild type strain YPH499 (*MAT*a *ura3-52 lys2-801_amber ade2-101_ochre trp1-*Δ*63 his3-*Δ*200 leu2-*Δ*1*)^46^. For overexpression of Dre2, the *DRE2* gene was subcloned into the pRS425 multi-copy plasmid 47. For expression of an IMS-directed version of Dre2 (*ims*Dre2), the sequences encoding the IMS-targeting sequence of Mia40 (residues 1 to 70) and the entire Dre2 protein were cloned into the plasmid pRS314 under control of the *MIA40* promoter 46. Deletion strains, Δ*glr1* and Δ*por1*, were generated by replacement of the genomic reading frame with a *kanMX4* cassette 48. Homologous recombination was verified by PCR. To deplete Mia40, a strain was used in which Mia40 expression was placed under control of a galactose promotor 27. Strains were grown in YP (1 % yeast extract, and 2 % peptone) medium with 2% glucose or galactose as carbon sources at 30°C, respectively. For all experiments using plasmid-containing strains, cells were grown in synthetic medium (0.17% yeast nitrogen base, 0.5% (NH_4_)_2_SO_4_, pH 5.5) containing all amino acids except for the auxotrophic markers and supplemented with 2% glucose or galactose at 30°C, respectively.

### Preparation of yeast mitochondria

Mitochondria were isolated as described 49. For isolation of cytosolic and mitochondrial fractions, the same protocol was used, however, bovine serum albumin was omitted from the homogenization buffer. The post-mitochondrial supernatant was cleared by ultracentrifugation (20 min, 100,000 x g) and used as cytosolic fraction. ^35^S-labeled precursor proteins (Dre2, *ims*Dre2, Oxa1, Tim9) were synthesized *in vitro* after subcloning of the reading frames into pGEM4 plasmids using reticulocyte lysate according to the protocol of the manufacturer (Promega, Mannheim, Germany). The import reactions and co-immunoprecipitation with Mia40-specific antibodies were performed as described previously 21,27,28. For the experiment shown in Fig. 6C, the radiolabeled proteins were precipitated with ammonium sulfate and denatured by 8 M urea, 10 mM EDTA, 5 mM β-mercaptoethanol, 50 mM Hepes pH 7.4 21. The import buffer and conditions for this experiment were as described in Banci *et al*., 2011 21.

### Localization of Dre2 and imsDre2

Mitochondrial sublocalization was performed by hypoosmotic swelling and digitonin fractionation. For hypoosmotic swelling, 50 μg mitochondria were incubated either in SH buffer (0.6 M sorbitol, 20 mM Hepes pH 7.4) or in 20 mM Hepes pH 7.4 for 20 min on ice in the absence or presence of 100 μg/ml proteinase K and/or 100 μg/ml trypsin. Protease digestion was stopped by the addition of 2 mM PMSF and/or 1 mg/ml soy bean trypsin inhibitor. Mitochondria were pelleted by centrifugation, washed with SH buffer containing 150 mM potassium chloride, and resuspended in 25 μl Laemmli buffer. For digitonin fractionation, 60 μg mitochondria were mixed with increasing concentrations (0 - 0.08 % w/vol) of digitonin and incubated on ice for 3 min. Subsequently, samples were diluted 1:15 in SH-buffer containing 100 μg/ml proteinase K. After incubation on ice for 20 min, protease digestion was stopped by addition of 2 mM PMSF. Mitochondria were reisolated by centrifugation at 20,000 x g for 10 min at 4°C and resuspended in 25 μl Laemmli buffer.

### Determination of the redox state of Dre2 in whole cells

Yeast cells were grown at 30°C to logarithmic phase. Four samples were adjusted to 1 ml with an OD_600_ of 2. For one of the samples (inverse shift), the cells were treated with 50 mM NEM for 15 min at 30°C. Cells of all four samples were pelleted, dissolved in 12% TCA and sonicated (21 times for 1 s). In two of the samples (inverse shift and maximum shift), the cells were now treated with modification buffer (80 mM Tris pH 7 (HCl), 10 % glycerol, 2 % SDS, bromocresol blue) containing the thiol-free reductant 10 mM tris(2-carboxyethyl)phosphine (TCEP) for 20 min at 96°C. Subsequently, 15 mM methyl-PEG-24-maleimide (mPEG-24, Thermo Scientific) was added in order to modify all reduced cysteines. To assess the *in vivo* redox state one sample was directly treated with modification buffer containing mPEG-24. As unmodified control, one sample was mock-treated with modification buffer without mPEG-24. All samples were incubated for 1 h in the dark and analyzed by Western blotting.

### Determination of the redox state of Dre2 in isolated mitochondria

Mitochondria were incubated with or without diamide or TCEP in SH buffer for 15 min at 30°C as described above. After TCA treatment samples were modified with mPEG-24 for 1 h in the dark and analyzed by Western blotting.

### *In vitro* protein oxidation with recombinant Mia40 and Mia40^SPS^

Expression and purification of recombinant Mia40 and Mia40^SPS^ was performed as described 28. The radioactive substrates Tim9, Dre2 and *ims*Dre2 were incubated with 35 μM recombinant Mia40 or Mia40^SPS^ at 20°C. After different reaction periods, samples were TCA-precipitated, resuspended in modification buffer and incubated with 15 mM methyl-PEG-12-maleimide (mPEG-12) for 1 h at 25°C. Samples were analyzed by SDS-PAGE and autoradiography.

## SUPPLEMENTAL MATERIAL

Click here for supplemental data file.

All supplemental data for this article are also available online at http://microbialcell.com/researcharticles/protein-oxidation-in-the-intermembrane-space-of-mitochondria-is-substrate-specific-rather-than-general-2/.
