# Supplementary material for: Protein oxidation in the intermembrane space of mitochondria is substrate-specific rather than general
Source: Microb Cell. 2014 Mar 3;1(3):81–93. doi: 10.15698/mic2014.01.130 (PMC5349226; doi:10.15698/mic2014.01.130)
Supplement: Supplementary file 1 [file mic-01-081-s01.pdf]

Supplemental Information

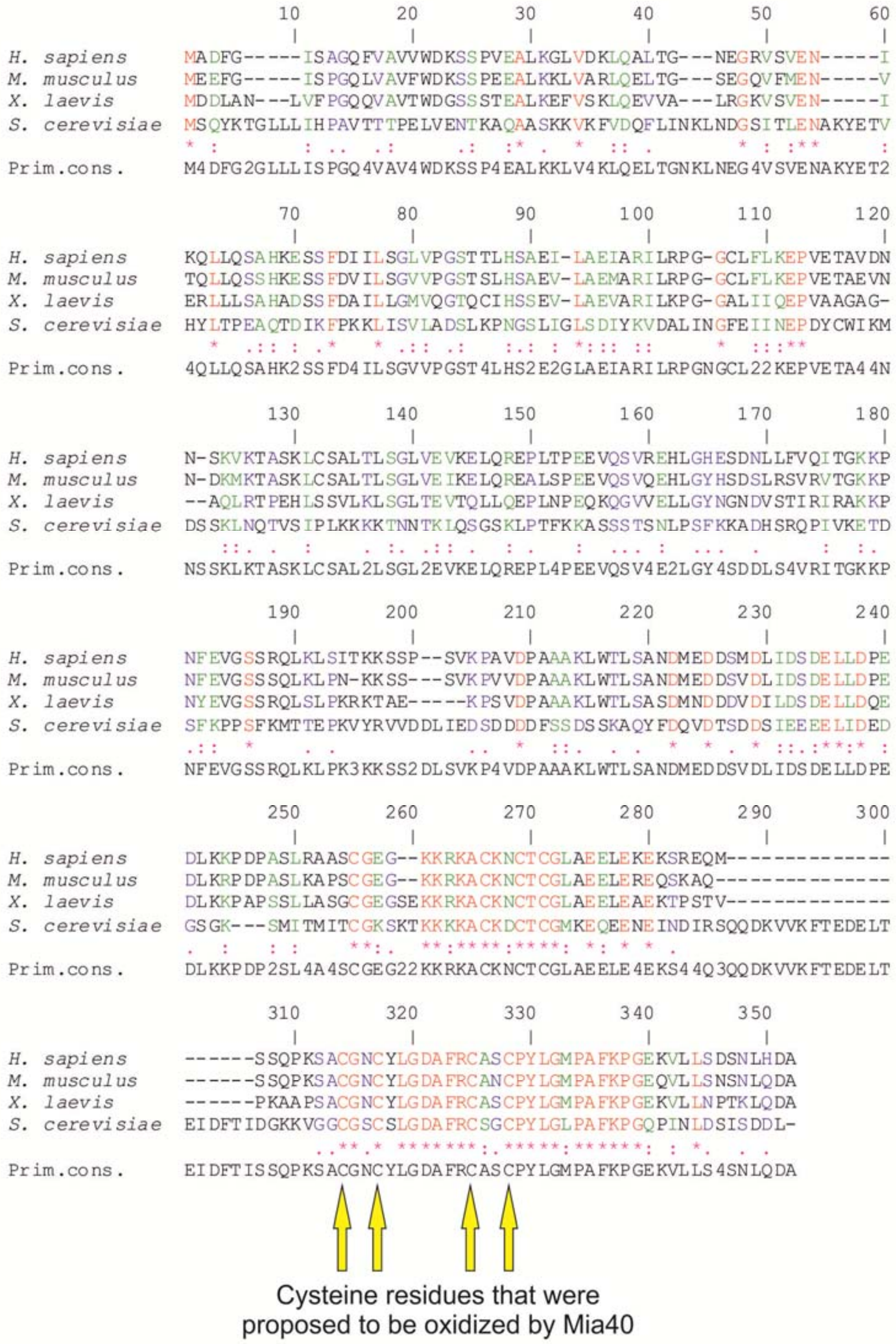

Fig. S1: Sequence alignment of members of the anamorsin/Dre2 family. The following sequences were aligned by use of the CLUSTAL W algorithm [1]: *Homo sapiens* NP_064709.2, *Mus musculus* NP_598902.1, *Xenopus laevis* NP_001164545.1, *Saccharomyces cerevisiae* P36152.1. The cysteine residues that were proposed to be oxidized by Mia40 in the conserved C-terminal region are indicated by arrowheads.

1. Thompson JD, Higgins DG, and Gibson TJ (1994). CLUSTAL W: improving the sensitivity of progressive multiple sequence alignment through sequence weighting, position-specific gap penalties and weight matrix choice. **Nucleic Acids Res.** 22, 4673-4680.
